# Lateral costal artery as a culprit for the steal phenomenon after coronary artery bypass grafting: a case report and review of the literature

**DOI:** 10.1186/s13019-024-03273-x

**Published:** 2025-01-07

**Authors:** Michal Trebišovský, Marián Homola, Adrián Kolesár, Štefan Lukačin, Anton Bereš

**Affiliations:** 1 Department of Heart Surgery, East Slovak Institute for Cardiovascular Diseases, Ondavská 8, Košice, 040 12 Slovakia; 2 Medical Faculty, Pavol Jozef Šafárik University, Trieda SNP 1, Košice, 040 11 Slovakia

**Keywords:** Left internal mammary artery, Coronary artery bypass grafting, Cardiac surgery, Lateral costal artery, Revascularization

## Abstract

**Background:**

The left internal thoracic artery (LITA) has been widely accepted as the standard for revascularizing the left anterior descending artery during coronary artery bypass grafting (CABG) surgery. However, in 10–20% of cases, the LITA may lead to unsecured side branches to the chest wall, particularly the lateral costal artery (LCA), potentially resulting in postoperative chest angina.

**Case presentation:**

We report the case of a 58-year-old patient who experienced persistent angina eight months after having undergone coronary artery bypass grafting (CABG) due to the steal phenomenon caused by a thick lateral costal artery (LCA). The LCA was found to be 2/3 the diameter of the left internal thoracic artery (LITA) with the decision to obliterate the LCA. Following LCA obliteration, the patient’s exertional angina was resolved.

**Conclusions:**

LCA may pose a potential issue in terms of coronary steal after CABG. Understanding the anatomy of the LITA with LCA variation and widening the opening of the pleura may be beneficial in preventing postoperative steal in selected cases.

**Supplementary Information:**

The online version contains supplementary material available at 10.1186/s13019-024-03273-x.

## Background

Ever since Loop et al. first published their findings in 1986, the left internal thoracic artery (LITA) has been widely accepted as the standard for revascularizing the left anterior descending artery during coronary artery bypass-grafting (CABG) surgery in patients with coronary heart disease [1]. Generally, arterial grafts have shown better long-term outcomes compared to venous grafts after CABG. As a result, the benefits of LITA harvesting have been firmly established in adult cardiac surgery. Despite its benefits, the LITA may give rise to unsecured side branches to the chest wall in 10–20% of cases, with the most significant being the lateral costal artery (LCA) due to its larger diameter. As a result, it may cause steal phenomenon due to competitive blood flow through these unsecured side branches, potentially resulting in postoperative chest angina [2, 3].

## Case presentation

A 58-years-old male patient (BMI 31 kg/m^2^) with a history of ischemic heart disease, hypertension, and smoking presented for elective coronary artery bypass grafting (CABG) due to stable angina. He has been suffering from hereditary hemochromatosis for 30 years, which has required treatment through venesections every three months. His preoperative coronarography indicated significant triple vessel disease: a 20% stenosis in the left main (LM) artery, a chronic total occlusion (CTO) in the middle part of the left anterior descending artery (LAD) with a thrombolysis in myocardial infarction flow score I (TIMI I), and 70% stenosis in the left circumflex (RCx) artery confirmed by an Instantaneous Wave-Free Ratio (iFr) of 0.86, and a 90% stenosis in the right coronary artery with a CTO (TIMI O) (Supplemental Video). According to coronarography, LAD extended to the apex. Preoperative echocardiography showed left ventricular ejection fraction of 65% without other notable pathologies regarding function of the right heart, valves and no signs of pulmonary hypertension.

In April 2022, patient underwent elective CABG with cardiopulmonary bypass. After opening the chest, standard cannulation for cardiopulmonary bypass was initiated. Blood cardioplegia was administered, and the heart was arrested as usual. Then, left internal thoracic artery (LITA) was connected to left anterior descending (LAD) artery and vena saphena magna (VSM) to posterior descending artery (PDA). Intraoperatively, inner diameter of LAD was fully patent for a 1 mm coronary probe. The surgery went well without any significant complications. The patient was transferred to the ICU without any inotropic or vasopressor support and was extubated within 3 h after the surgery. On the third day after the surgery, there was an increase in C-reactive protein (175 mg/L) and procalcitonin levels (0.04 ug/L) with white blood cell count of 13.3 × 10^9^/L treated with combination cefotaxime and ciprofloxacin as per the local protocol. Eventually, the patient was discharged home on the 10th postoperative day with negative procalcitonin levels and a well-appearing sternal wound.

**Fig. 1:**
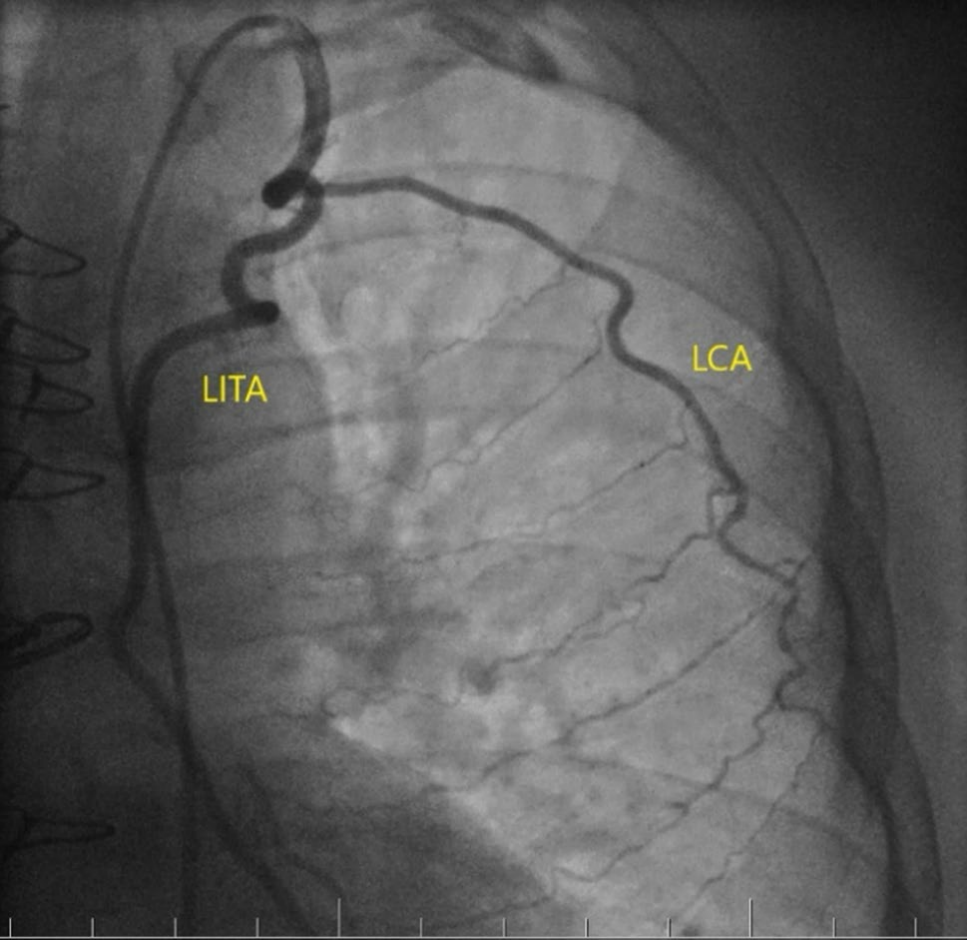
Coronary angiography showing large LCA originating from LITA after 15 mm of its offset from the subclavian artery. LCA – Lateral costal artery, LITA – left internal mammary artery

**Fig. 2:**
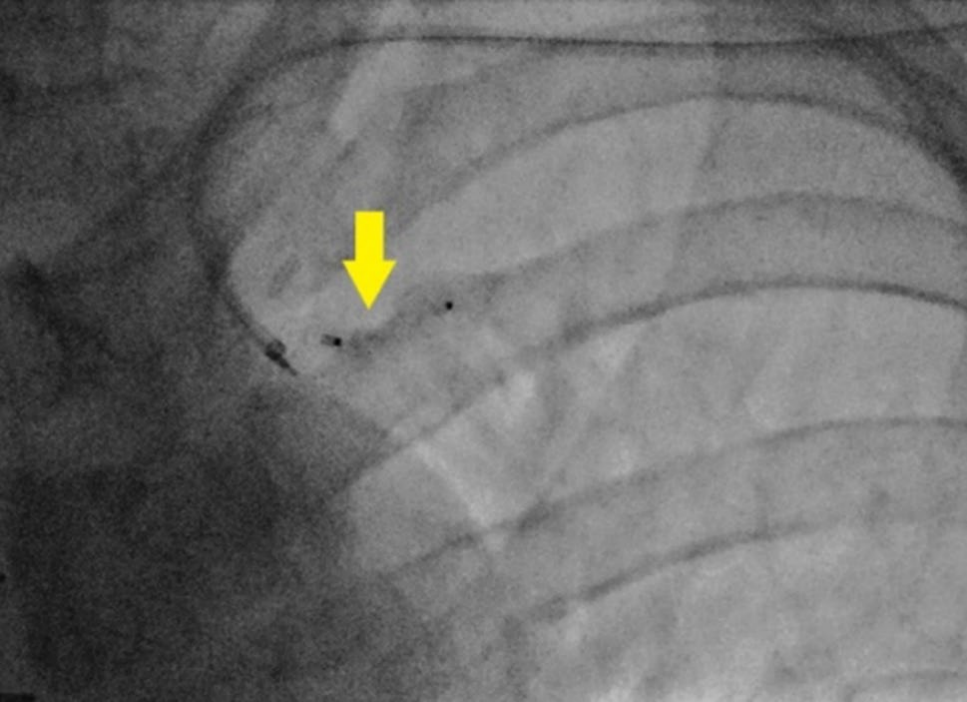
Coronary angiography showing occluded LCA with ceased flow through LCA

After eight months post-surgery, the patient began experiencing persistent chest pain and dyspnoea during physical activity. He was readmitted to our hospital for further investigation. An ECG did not show any signs of myocardial ischemia and an echocardiogram did not reveal any new regional wall motion abnormalities. However, during coronary angiography, a lateral costal artery (LCA) was found to be abnormally large, extending up to the sixth intercostal space (ICS) and forming connections with intercostal arteries. The LCA was found to have 2/3 of the diameter of the LITA (Fig. 1). It was suspected that the LCA might be causing a coronary steal phenomenon, redirecting a significant portion of the blood flow away from the myocardium toward the lateral chest wall. It was decided to obliterate the LCA using a vascular closure device (Abott-Amplatz Vascular Plug II closure device 4 × 6 mm, Plymouth-MN, USA), and the procedure was successfully performed (Fig. 2).

After closing the LCA, the patient’s exertional angina resolved. He was followed up for the next year reporting no angina on exertion.

## Discussion and Conclusions

Harvesting LITA can be challenging in obese patients or those with pleural adhesions due to previous lung diseases or trauma such as rib fractures. These conditions can make LITA harvest difficult. Two basic techniques are commonly used for LITA grafts: pedicled and skeletonized grafts. Both methods have their advantages and disadvantages, and the choice usually depends on the surgeon’s preference. Regardless of the technique chosen, the harvest stops near the subclavian vein and the level of the first rib where the largest side branch can be present.

Evidence suggests that keeping the pleura intact may benefit patients in terms of improved postoperative pulmonary mechanics as improvement spirometry values as forced vital capacity (FVC%) and forced expiratory volume in the first second (FEV1%). However, not opening the pleura can prevent the identification and securing large side branches (i.e. LCA), but also has fewer complication with regard to lung atelectasis and pleural effusion [4].

Anatomy of the LITA may vary. LITA originates from the first part of the subclavian artery, approximately 2 cm above the sternoclavicular junction, opposite to the origin of the thyrocervical trunk. LITA directly rises from the left subclavian artery in 70% of cases while the remaining 30% originates from the common trunk [5]. Later in its course, it descends dorsally and laterally to the sternum at the level of 1st to 6th rib where it terminates as musculophrenic and superior epigastric arteries. The LITA gives pericardiophrenic, thymic, sternal, anterior costal, perforating and LCA branches. In a study by Bauer et al. involving 262 CABG patients, it was found that the LITA has large side branches in 9% of cases and has an atypical location in 1% of cases [6]. In the literature, the incidence of LCA is reported to be in 10–30% of cases (Table 1).

**Fig. 3:**
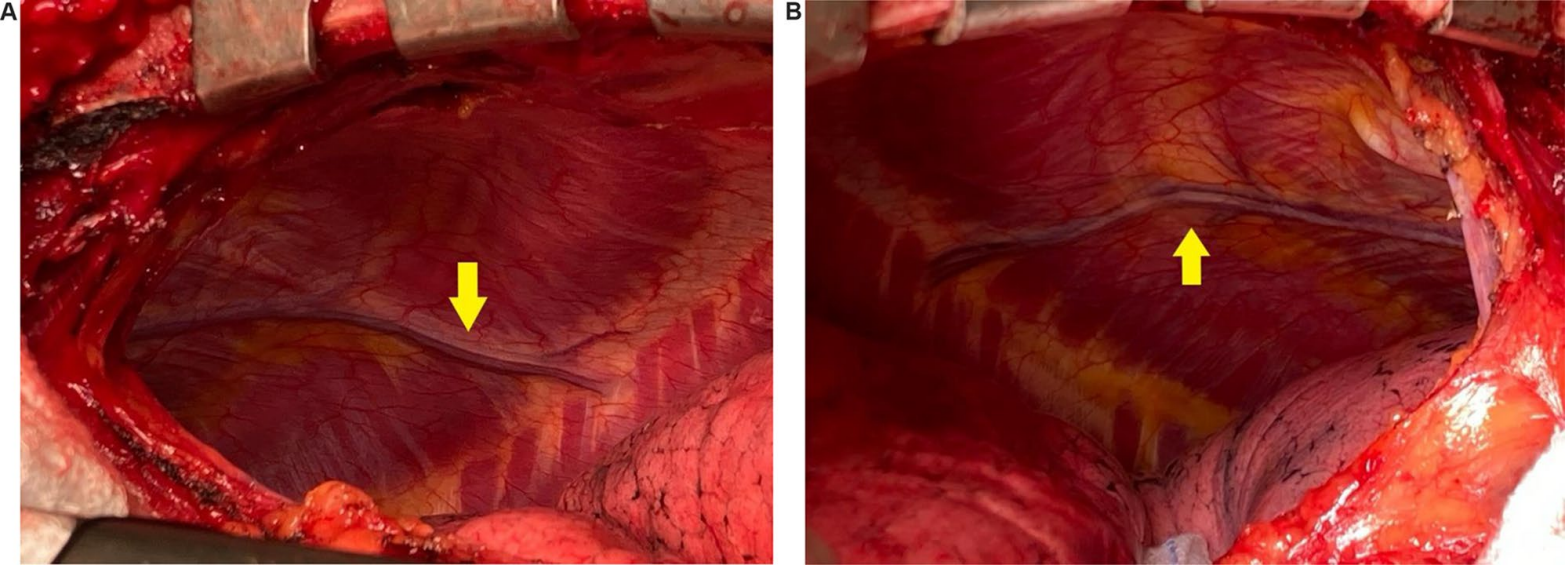
**A** Left pleural view showing large LCA. **B** Right pleural view showing large LCA

**Table 1:** Incidence of LCA in the literature, * cadaverous findings, LCA – lateral costal artery, n = number of subjects

Author	Year	*n*	%
Calafiore et al. [7]	1990	150	11%
Henriquez-Pino et al. [5]	1997	100*	10% unilateral LCA 5% bilateral LCA
Sutherland et al. [8]	2000	103	5% unilateral LCA 25% bilateral LCA
Nakawaza et al. [9]	2022	973	19%

The LCA stems from LITA and runs parallel and distal to the LITA from which it originates. It follows a descending path toward the anterior chest wall at the level of the medial axillary line, extending from the 2nd to the 6th ICS. It is accompanied by two satellite veins that create horizontal bridging anastomoses, forming a single vein that connects with the intercostal veins at the 6th ICS [10].

The LCA is clinically important for cardiac and for thoracic surgery for several reasons. First, LCA can be the cause of a steal phenomenon after harvesting and performing LITA to LAD during CABG surgery where steal phenomenon may result in systolic flow diversion and not a true coronary flow steal, because the arterial flow to chest wall is predominantly systolic opposed to coronary flow which is diastolic [2]. Second, the course and anatomical localization, as well as the length of the LCA artery, may increase the risk of artery puncture when draining pneumothorax or fluidothorax, regardless of whether the puncture is performed in the safety triangle. This is due to LCA extending as far as 5th ICS in 23% of patients [9]. Third, its importance may lie as additional arterial graft forming a native Y-graft arterial conduit for total-artery coronary revascularization as described by Hartman et al. [11].

Interestingly, only 59% of the cases in Nakazawa et al. study had bilaterally missed lateral costal vessels, and lateral costal vessels were present unilaterally in 33% (right 22% and left 11%) and bilaterally in 7% of the cases [9].

Due to unique anatomic approach, harvesting LITA through left anterior thoracotomy has higher rates to unrecognized and therefore unsecured LCA. However, endoscopic harvesting in minimal invasive cardiac surgery can be beneficial for reaching pleural cupula to visualise LCA [9, 12].

Given the above, option to prevent postoperative ischemic complications related to the steal phenomenon is to perform a wide opening of the pleura, which would help to detect unrecognized LCA. Our centre’s experience suggests that several cases of LCA have been accidentally detected with wide pleural openings during LIMA harvest. Although the true prevalence of large LCA is unknown, our centre experiences at least 6 cases per year which can be both, unilateral or bilateral (Fig. 3).

To conclude, LCA may present a potential problem in terms of coronary steal after the CABG. Whether routine subclavian angiography as part of coronary angiography could detect large LCA before elective cardiac surgery remains unclear. Knowledge of LITA anatomy with LCA variation as well as wide-opening the pleura may be beneficial in selected cases.

## Electronic supplementary material

Below is the link to the electronic supplementary material.

Supplementary Material 1: Video file: Complete epicardial filling by collateral circulation form RCA and RMSI) with CC2 side branches from LAD is visible and corresponds to Rentrop 3 classification

## Data Availability

No datasets were generated or analysed during the current study.

## Abbreviations

BMI: Body mass index
CABG: Coronary artery bypass grafting
CTO: Chronic total occlusion
ICS: Intercostal space
LAD: Left anterior descending
LCA: Lateral costal artery
LITA: Left internal thoracic artery
LM: Left main coronary artery
TIMI: Thrombolysis In Myocardial Infarction
RCx: Circumflex artery
CC2: Collateral circulation

## References

[1] Loop FD, Lytle BW, Cosgrove DM, Stewart RW, Goormastic M, Williams GW, Golding LA, Gill CC, Taylor PC, Sheldon WC, et al. Influence of the internal-mammary-artery graft on 10-year survival and other cardiac events. N Engl J Med. 1986;314(1):1–6. 3484393 10.1056/NEJM198601023140101

[2] Kern MJ. Mammary side branch steal: is this a real or even clinically important phenomenon? Ann Thorac Surg. 1998;66(6):1873–5. 9930462 10.1016/s0003-4975(98)01054-6

[3] ALEXANDER WF. The course and incidence of the lateral costal branch of the internal mammary artery. Anat Rec. 1946;94:446. 21020572

[4] Rezk ME, Elgazzar MA, Abo Youssef SM, Emeraa AS, Elkafoury AE, Moussa HH. Open Versus Closed Pleura Internal Mammary artery harvesting and early pulmonary function after coronary artery bypass grafting. Heart Lung Circ. 2020;29(9):1412–7. 31786114 10.1016/j.hlc.2019.09.014

[5] Henriquez-Pino JA, Gomes WJ, Prates JC, Buffolo E. Surgical anatomy of the internal thoracic artery. Ann Thorac Surg. 1997;64(4):1041–5. 9354524 10.1016/s0003-4975(97)00720-0

[6] Bauer EP, Bino MC, von Segesser LK, Laske A, Turina MI. Internal mammary artery anomalies. Thorac Cardiovasc Surg. 1990;38(5):312–5. 2264041 10.1055/s-2007-1014041

[7] Calafiore AM, Contini M, Iacò AL, Maddestra N, Paloscia L, Iovino T, Di Mauro M. Angiographic anatomy of the grafted left internal mammary artery. Ann Thorac Surg. 1999;68(5):1636–9. 10585033 10.1016/s0003-4975(99)00835-8

[8] Sutherland FW, Desai JB. Incidence and size of lateral costal artery in 103 patients. Ann Thorac Surg. 2000;69(6):1865–6. 10892938 10.1016/s0003-4975(00)01311-4

[9] Nakazawa S, Kawatani N, Obayashi K, Ohtaki Y, Ito T, Yajima T, Shirabe K. Clinical and anatomical features of the lateral costal artery and vein. Sci Rep. 2022;12(1):10589. 35732684 10.1038/s41598-022-14318-3 PMC9217911

[10] Emilio F-C, Oscar I-H. Echeverría-M. Mark. Lateral-bilateral Costal Branch (R. Costalis Lateralis): a clinically relevant Anatomical Variation. Int J Morphol [Internet]. 2017 Dec [cited 2024 Aug 20]; 35(4): 1512–6.

[11] Hartman AR, Mawulawde KI, Dervan JP, Anagnostopoulos CE. Myocardial revascularization with the lateral costal artery. Ann Thorac Surg. 1990;49(5):816–8. 2339940 10.1016/0003-4975(90)90033-3

[12] Vural Ü, Aglar AA, Sahin S, Kizilay M. Lateral costal artery: clinical importance of an accessory thoracic artery. Braz J Cardiovasc Surg. 2018 Nov-Dec;33(6):626–30. 10.21470/1678-9741-2017-0252 PMC6326454 30652753

